# The Distressed Brain: A Group Blind Source Separation Analysis on Tinnitus

**DOI:** 10.1371/journal.pone.0024273

**Published:** 2011-10-06

**Authors:** Dirk De Ridder, Sven Vanneste, Marco Congedo

**Affiliations:** 1 Brai^2^n, TRI & Department of Neurosurgery, Antwerp University Hospital, Antwerp, Belgium; 2 Team ViBS (Vision and Brain Signal Processing), GIPSA-lab, National Center for Scientific Research, Grenoble University, Grenoble, France; University of Bern, Switzerland

## Abstract

**Background:**

Tinnitus, the perception of a sound without an external sound source, can lead to variable amounts of distress.

**Methodology:**

In a group of tinnitus patients with variable amounts of tinnitus related distress, as measured by the Tinnitus Questionnaire (TQ), an electroencephalography (EEG) is performed, evaluating the patients' resting state electrical brain activity. This resting state electrical activity is compared with a control group and between patients with low (N = 30) and high distress (N = 25). The groups are homogeneous for tinnitus type, tinnitus duration or tinnitus laterality. A group blind source separation (BSS) analysis is performed using a large normative sample (N = 84), generating seven normative components to which high and low tinnitus patients are compared. A correlation analysis of the obtained normative components' relative power and distress is performed. Furthermore, the functional connectivity as reflected by lagged phase synchronization is analyzed between the brain areas defined by the components. Finally, a group BSS analysis on the Tinnitus group as a whole is performed.

**Conclusions:**

Tinnitus can be characterized by at least four BSS components, two of which are posterior cingulate based, one based on the subgenual anterior cingulate and one based on the parahippocampus. Only the subgenual component correlates with distress. When performed on a normative sample, group BSS reveals that distress is characterized by two anterior cingulate based components. Spectral analysis of these components demonstrates that distress in tinnitus is related to alpha and beta changes in a network consisting of the subgenual anterior cingulate cortex extending to the pregenual and dorsal anterior cingulate cortex as well as the ventromedial prefrontal cortex/orbitofrontal cortex, insula, and parahippocampus. This network overlaps partially with brain areas implicated in distress in patients suffering from pain, functional somatic syndromes and posttraumatic stress disorder, and might therefore represent a specific distress network.

## Introduction

At some point in life most people experience a sound in their ears or head although no external sound is present [1]. This has been related to listening to loud music[2], sudden sensorineural hearing loss[3], use of medication[4], trauma[5] or other causes. Typically, this sensation is reversible and subsides approximately between a few seconds to a few days. The early explorers of Africa titrated the dose of quinine to the reversible presence of a phantom sound, as was done for aspirin in the treatment for rheumathoid arthritis and gout[6]. This phantom sound is also called tinnitus. To date, no FDA approved pharmacological treatment exists for this auditory phantom phenomenon [7].

In an adult population 10 to 15% of the population perceives tinnitus chronically and about 6 to 25% of the affected people report interference with their daily living, as tinnitus can cause a considerable amount of distress, involving sleep deprivation[8], [9], depression[10], annoyance, cognitive problems[11], and work impairment [1], [9], [12], [13], [14].

Therefore, tinnitus is usually evaluated for both its intensity or loudness by tinnitus matching or VAS scores and for its annoyance or distress, using validated tinnitus questionnaires. One of the surprising findings in tinnitus research is that the perceived tinnitus intensity as determined by tinnitus matching correlates poorly with the associated distress [15]–[16], suggesting that separable networks might be involved in both aspects of tinnitus. This is clinically well known from the 1930's and 1940's when frontal lobotomies were performed for the treatment of tinnitus, resulting in unchanged tinnitus intensity but markedly decreased tinnitus annoyance [17], [18].

Magnetoencephalography (MEG) studies have demonstrated that tinnitus is correlated to decreased alpha [19] and associated increased gamma band activity in the contralateral auditory cortex [20], [21]. Furthermore, the amount of contralateral gamma band activity as estimated by EEG current density correlates with the perceived intensity of the phantom sound.

On the other hand, a recent study, using LORETA source localization in EEG, revealed that distress in tinnitus patients is related to increased beta activity in the dorsal part of the anterior cingulate cortex (ACC) and the amount of distress correlates with an alpha activity in several brain areas such as the amygdala, ACC, insula and parahippocampus [22]. A MEG study further showed that long-range coupling between frontal, parietal and cingulate brain areas in ‘alpha and gamma networks’ is related to tinnitus distress [23]. Due to the low spatial resolution of this MEG study (based on a coarse inverse solution) it cannot be deduced whether the frontal area also incorporates the anterior insula found in the source localization EEG study. The distress in tinnitus patients also correlates with an increase in incoming and outgoing connections in the gamma band in the prefrontal cortex, the orbitofrontal cortex and the parieto-occipital region [24]. The available spectral EEG and network MEG literature suggests that the increased spontaneous resting state activity and connectivity present in tinnitus distress is the result of a ‘distress network’ separable from a tinnitus intensity network. Thus the question arises whether the increased alpha activity in the amygdala, anterior cingulate cortex, parahippocampus and insula and the beta activity in the dorsal anterior cingulate form one ‘distress network’ of functionally interconnected areas, as one separable component of multiple overlapping tinnitus networks each defining a specific tinnitus characteristic such as laterality [25], tinnitus type (pure tone vs noise-like tinnitus) [26] etc. Two main data analysis approaches have been used to study functional connectivity, i.e., the correlations between spatially remote neurophysiological events in resting state networks by fMRI [27]; a seed-based connectivity analysis and independent component analysis (ICA). The latter is currently enjoying increasing popularity thanks to its complete data-driven nature [28], [29], [30]. Another source separation method similar to ICA has recently been extended to group analysis of resting state EEG, test-retesting two independent EEG databases in normal population. This resulted in the discovery of seven replicable groups blind source separation (BSS) components explaining about 92% of the variance [31](Table 1). As any other source separation method of this family, the BSS approach we use decomposes the whole EEG in a number of elementary statistically independent components, each one characterised by its time course and spatial pattern, therein used as input to source localization by the sLORETA inverse solution [32].

**Table 1:** Anatomical Structures and Brodmann Areas of the sevec normative independent components (IC) [67].

IC1	Anterior Cingulate (BA 23/24/32/33/25), Insula (BA 13), Middle/Superior Frontal Gyrus and Paracentral Lobule (BA 4/5/6), Parahippocampal/Subcallosal Gyrus (BA 28/34/35/36)
IC2	Cuneus/Precuneus/ (BA 7/31/18/19/), Post-central gyrus (BA 3/4/5), Superior Parietal and Paracentral Lobule (BA 5/7), Posterior Cingulate Gyrus (BA 23/31)
IC3	Cuneus/Precuneus/ (BA 30/31/7), Right superior parietal lobule (BA 7), Posterior Cingulate (BA 30), Lyngual/Parahippocampal Gyrus (BA 18/19/30), Right Fusiform Gyrus (BA 19)
IC4	Cuneus/Precuneus/Posterior Cingulate (BA 23/30/31), Lyngual Gyrus/Fusiform Gyrus/Middle and Inferior Occipital Gyrus (Occipital Pole) (BA 17/18/19)
IC5	Anterior Cingulate (BA 24/25/32), Medial Frontal Gyrus (BA 32/9/10/11), Rectal/Orbital Gyrus (BA 11/47), Inferior Frontal Gyrus (BA 47), Parahippocampal Gyrus (BA 28/34)
IC6	Medial Frontal/Rectal Gyrus/Anterior Cingulate (BA 11, 25), Middle Frontal Gyrus (BA 11), Inferior Frontal Gyrus (BA 47), Parahippocampal Gyrus (BA 28/34), Insula (BA 13)
IC7	Post-central Gyrus (BA 1/2/3), Supramarginal Gyrus/Inferior Parietal Lobule (BA 40), Precentral Gyrus (BA 6), Cuneus/Precuneus (BA 17/18/19/31), Middle Occipital Gyrus (BA 18), Superior and Middle temporal Gyrus (BA 21/22/39/41), Insula (BA 13), Angular Gyrus (BA 39)

This study analyzes the group BSS networks in tinnitus and tinnitus distress, by comparing the resting state electrical activity of a very homogeneous group of tinnitus patients with controls and by comparing low and high distress, with the clinical groups not different for tinnitus type, tinnitus duration or tinnitus laterality (Table 1). This ‘functional network’ is further verified by performing a multivariate lagged coherence analysis between the brain areas defined by the group BSS results.

## Results

### Normative Group Blind Source Separation

**Figure 1:**
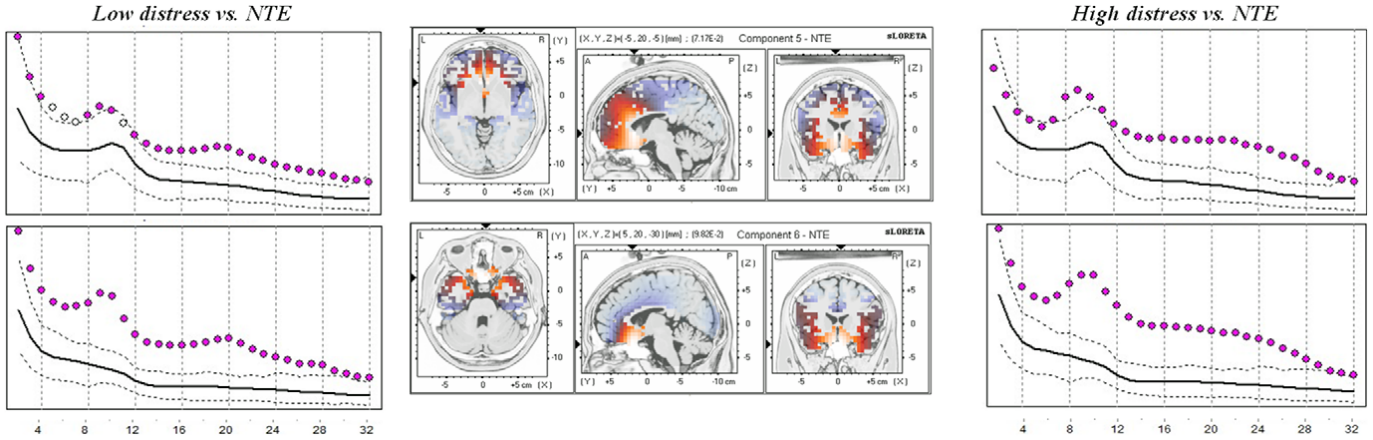
Comparison for the normative independent components IC5 and IC6. Left and right panels: Relative Power (arbitrary units) of components along frequencies in the range 2–32 Hz for low distress (left) and high distress (right) in tinnitus patients. Black solid and dotted lines represents the mean and 95% confidence intervals, respectively, for the normative group. Disks represent the mean of the clinical group. Pink disks flag a statistically higher power (p<0.01) in the relative mean power of the patients as compared to the normative database. Middle power: the sLORETA source localization of IC5 and IC6 (Congedo et al., 2010).

Comparisons for the components generated on the normative database and compared with the two tinnitus groups (low and high distress) revealed significant differences for the relative power (*p*<.01) for two of the seven components: IC5 and IC6. An overview is given in Figure 1. Table 1 specifies the Brodmann areas involved in each component. For both components more activity was revealed in all frequencies bands (delta, theta, alpha, beta and gamma) for both the low and high tinnitus distress in comparison to normative database. Since we analyzed relative power measures (with respect to the total power of the seven components) this shows that these two components are overall predominant in the EEG of the patients as compared to the normative group, irrespective to frequency.

A comparison between low and high distress for the different components revealed only a significant effect for component 6 for the frequency band 14–18 Hz (*t* = 2.57, *p*<.05) and 22–26 Hz (*t = *2.82, *p*<.05). No other component did obtain significance.

### Additional analysis

An additional analysis was conducted comparing the normative group with age-matched tinnitus patients (Table S1) for respectively low and high distress. Similar results were obtained as for the whole group and for the older tinnitus patients (Figures S1, S2).

For IC5, Visual inspection indicate that for the young tinnitusgroup had less delta, theta, alpha activity compared to the older tinnitus group for patients with low distress. However, there were no significant effect differences for the young and old group For patients with high distress, visual inspection indicicates that young tinnitus group had less delta and theta compared to the older tinnitus group. Again, no significant effect was obtained when comparing the young and old tinnitus group with high distress. Yet, both the low and high distress patients showed, independently of belonging to theyoung or old tinnitus group, significantly increased activity compared the normative database.

For IC6 visual inspection indicates a difference between theyoung tinnitus group in comparison to the older tinnitus group with high distress in delta and theta. However, a statistical comparison between both groups showed no significant differences. Both groups showed significantly increased activity in comparison to the normative database.

### Correlation analysis

Correlation analysis revealed a significant (p<0.05) positive correlation between the log-power of two components and the TQ-scores: a significant positive correlation was found in the alpha (8–12 Hz) and beta (12–16 Hz and 16–20 Hz) range for component 5 and in the alpha (8–12 Hz) and beta (12–16 Hz and 16–20 Hz as well as 22–26 Hz) range for component 6 (Table 2 and Figures S3, S4). Also after exclusion of potential outliers, correlations remained significant. No other component reached significance.

**Table 2:** Correlation analysis between TQ and BSS components.

	Frequencies	r
IC5	8–12 Hz	.28*
	12–16 Hz	.32**
	16–20 Hz	.24*
IC6	8–12 Hz	.25*
	12–16 Hz	.36**
	16–20 Hz	.26*
	22–26 Hz	.34**
Tinnitus IC4	8–12 HZ	.28*
	12–16 Hz	.28*
	16–20 Hz	.27*
	20–24 Hz	.30*

See additional figure in supplementary material (Figures S3S, S4, S5).

*p<.05;

**p<.01.

### Group Blind Source Separation on the Tinnitus Sample

**Figure 2:**
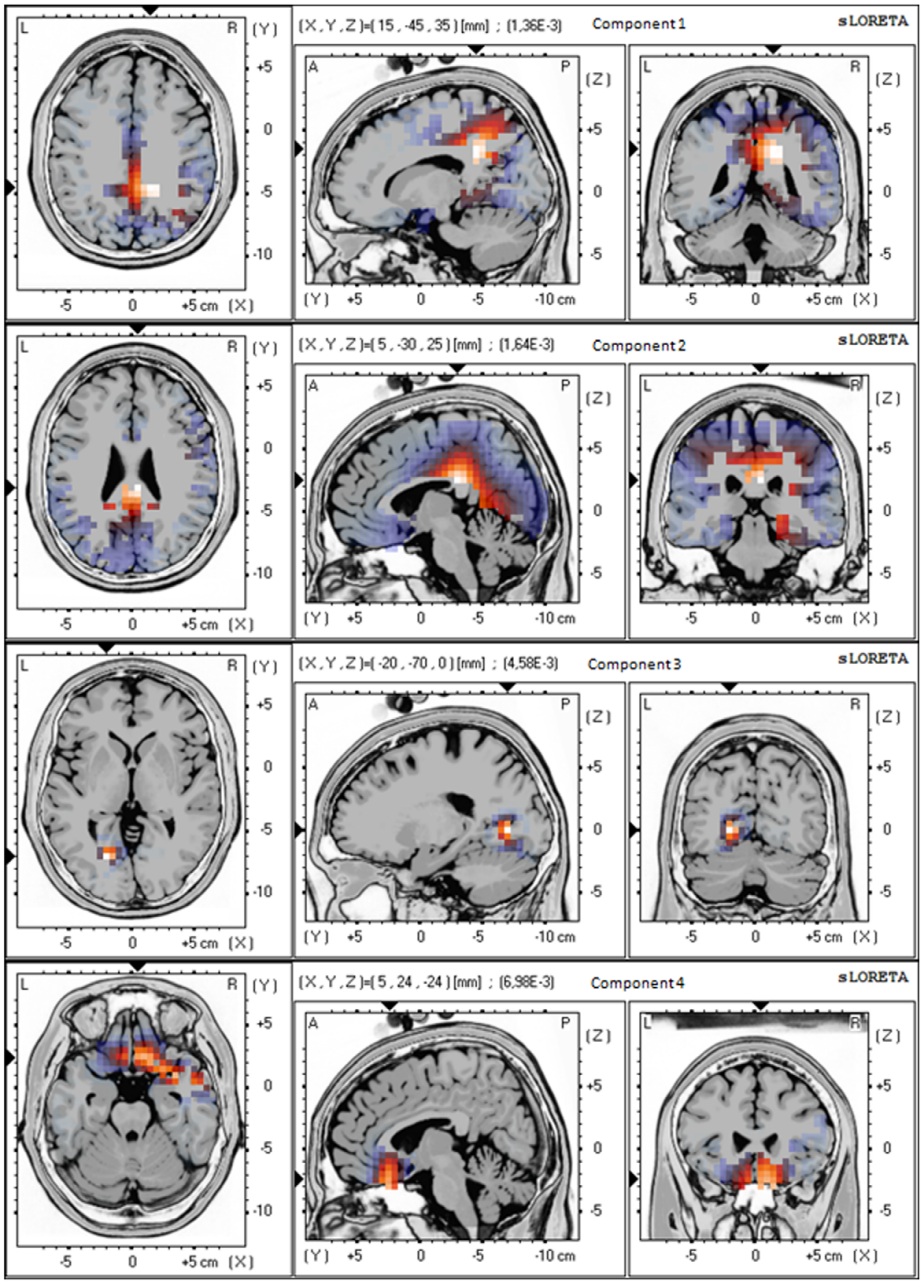
The four most energetic components obtained by applying group Blind Source Separation on the tinnitus group.

For the group BSS on the tinnitus group the Akaike information criterion (AIC) suggested to retain the four most energetic components, explaining 52% of the total variance (Figure 2). Component 1 and 2 showed increased activity in the posterior cingulate cortex (BA23, BA33), precuneus (BA7), retrosplenial posterior cingulate cortex (BA29, BA30), and subgenual anterior cingulate cortex (BA25). Component 3 revealed activity in parahippocampal area (BA19, BA30), while component 4 demonstrated activity in subgenual anterior cingulate cortex (BA25) extending into right inferior frontal gyrus (BA47). Significant positive correlations (p<0.05) were found between components obtained for the tinnitus group and the TQ-scores for alpha (8–12 Hz), beta (12–16 Hz, 16–20 Hz, 20–24 Hz) for component 4 (Table 2 and Figure S5). No other component did obtain significance.

### Multivariate Functional Connectivity Analysis

**Figure 3:**
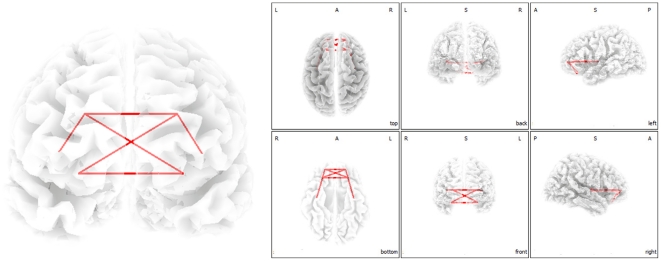
Phase synchronization analysis demonstrating increased functional connectivity within the region of interest of component 5 and 6 for 8–12 Hz for tinnitus patients in comparison to normative database.

**Figure 4:**
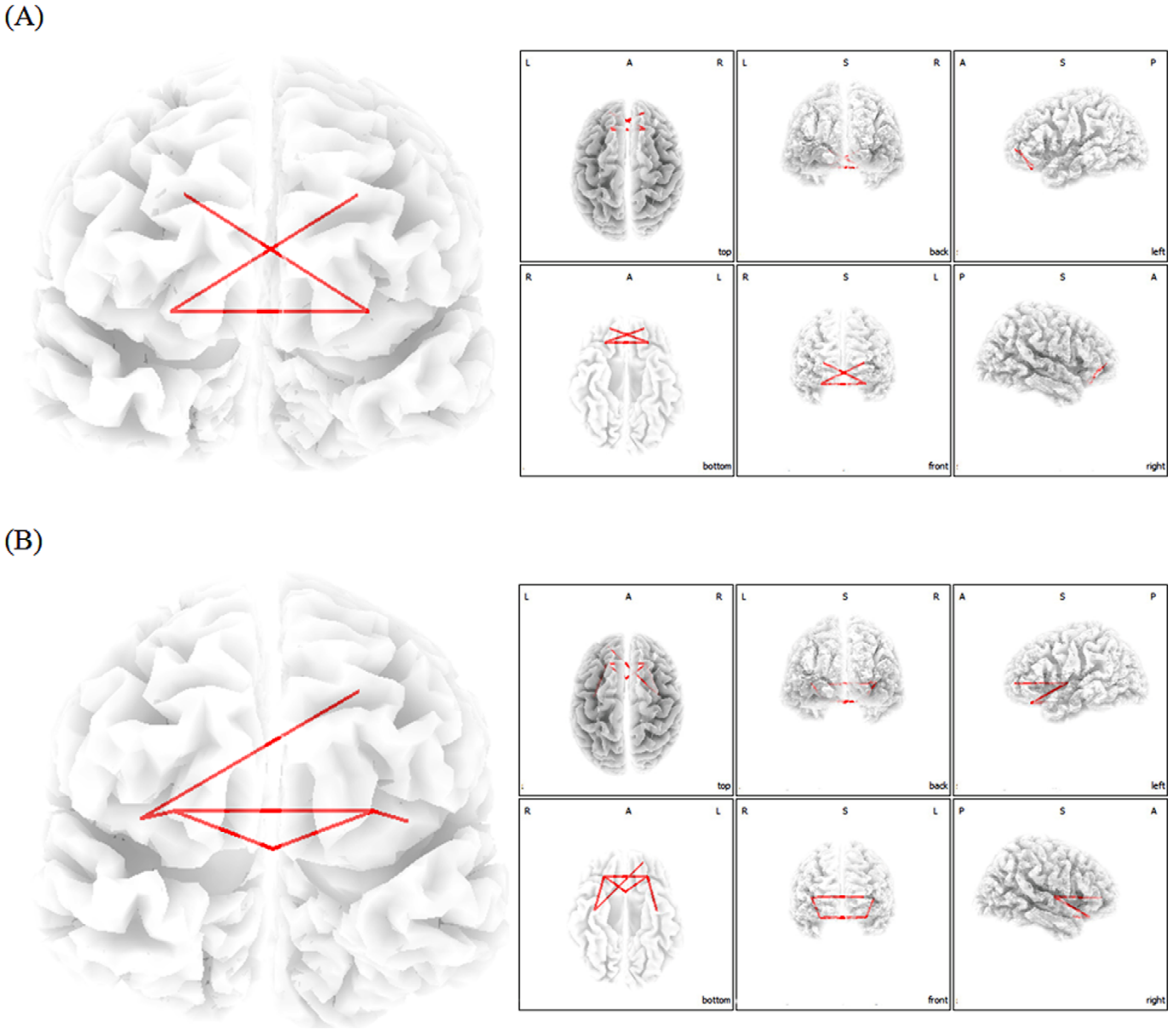
Phase synchronization analysis demonstrating increased functional connectivity within the region of interest of component 5 and 6 for 12–16 Hz (A) and 16–20 Hz (B) for tinnitus patients in comparison to normative database.

We verified the group BSS defined functional networks with a functional connectivity analysis evaluating lagged coherence between the areas defined by the BSS analysis. These additional analyses revealed an increased functional connectivity between the (para)hippocampus, subgenual anterior cingulate cortex, orbitofrontal cortex and the inferior frontal gyrus for alpha (8–12 Hz) (Figure 3) and for beta (12–16 Hz and 16–20 Hz) (Figure 4).

### Hearing loss

No significant correlation was found for hearing loss as measured by the loss in decibels (dB SPL) at the tinnitus frequency and the independent components.

## Discussion

Based on the Aikake information criterium tinnitus is characterized by at least four independent components, two of which are posterior cingulate based, one based on the subgenual anterior cingulate and one based on the parahippocampal area. The anterior cingulate has been implicated in emotional [33], attentional [34], reward [35] and executive [36] processing, whereas the posterior cingulate seems to be related more to cognitive and memory aspects of information processing [36].

The posterior based components found in this analysis might thus be related to cognitive and memory related aspects of the tinnitus percept, as the retrosplenial PCC (BA 29& 30) is implicated in auditory memory [37], [38] and the PCC is involved in cognitive aspects of auditory processing [39]. Activity in the precuneus and adjacent retrosplenial and posterior cingulate cortex has indeed been linked to successful retrieval from auditory (and visual) memory [40], [41]. The PCC/precuneus component has been proposed to exert a salience based cognitive auditory comparator function [39]. When the PCC component is deficient or less active, such as in tinnitus distress [22], this could reflect an incapacity of the PCC/precuneus to exert its salience based comparator function, pulling irrelevant auditory (tinnitus)sound from hippocampal memory [42], via dysfunctional parahippocampal auditory sensory gating [43], analogous to what has been proposed for auditory hallucinations [44].

The subgenual anterior cingulate (BA25) based independent component in tinnitus patients is similar to the IC5 and IC6 (Table 1) described in a normative database [31], and it is therefore interesting to compare these tinnitus related components to a normative database. IC5 and IC6 make up overlapping networks consisting of the subgenual ACC extending to the pregenual and dorsal ACC as well as the ventromedial and ventrolateral prefrontal cortex/orbitofrontal cortex, insula, and parahippocampal area. IC5 is more centered on the dorsal ACC, whereas IC6 is centered on the subgenual cingulate, extending into the orbitofrontal cortex and insula.

The comparison of mild and very severe distress in tinnitus and their comparison to a normative ICA EEG database yields both spatial and spectral information distinguishing distress in tinnitus patients from norms and distinguishing mild from severe distress in this patient group.

Two anatomically specified networks (components IC5 and IC6, Table 1) of the normative database yield significant distress related differences in tinnitus patients in comparison to controls and one of these components (IC6) separates tinnitus patients who suffer a lot from those who do not suffer or only suffer mildly. The fact that distress is a network property fits with a recent MEG study using network analysis demonstrating multiple hubs [24] in a large scale network involved in tinnitus distress [45].

The subgenual component found in the tinnitus group is the only component correlating to the tinnitus distress (Table 2), adding further data and confirming the concept that components 5 and 6 in the normative database constitute networks which can be involved in the generation of distress.

It has been recently proposed that tinnitus is the result of a deficient noise cancellation mechanism originating in the nucleus accumbens-subgenual cingulate area [46]. This area would modulate thalamocortical dysrhythmic activity via the reticular nucleus of the thalamus [46]. Thus stress could modulate this putative noise-cancelling mechanism, explaining potentially both the fact that many people attribute their tinnitus and pain to stress, and that distress often accompanies phantom perceptions [47].

The IC6, which differentiates between distressed and non-distressed patients, represents an emotion and autonomic nervous system binding network (Table 1, Figure 1). This component binds brain areas involved in tinnitus distress, as described in a smaller set of patients using a different technique [22]. In this study, source analyzed FFT spectral analysis of distress in comparison to no distress correlated with beta activity in the dorsal ACC, and the amount of distress correlated with alpha activity in the sgACC, insula, amygdala, and parahippocampal area, associated with a decrease in alpha activity in the PCC. FFT based functional connectivity, as analyzed by instantaneous coherence between the areas defined by the ICA analysis (IC5 and IC6) further reveals that the brain areas involved in distress [22], really form a functional network.

In these IC5 and IC6 networks increasing distress also correlates with increasing beta activity. The IC5 and IC6 related beta activity is consistent with a recent EEG study looking at spectral differences between high and low distress [22]. Increased beta activity is noted in tinnitus distress patients in comparison to the normative database in the anterior cingulated based IC5 and IC6 components [22].

The increased alpha activity in the subgenual ACC noted in that study[22] is also retrieved in this analysis, but only in the FFT on the subgenual component of the tinnitus, which correlates with the perceived amount of distress.

Severe distress such as in posttraumatic stress disorder is associated with increased beta activity especially over frontal and central areas (C3,C4,F3,F4) [48], [49]. The beta activity in the anterior cingulate-anterior insula network might therefore reflect the expression of an aspecific distress network, common to tinnitus patients and PTSD patients. Further arguments for the existence of such a non-specific distress network can be derived from the fact that pain distress [50], [51], distress in asthmatic dyspnea [52] and distress in functional somatic syndromes such as electro-sensitivity for mobile phones [53] as well as social rejection distress [54] also correlate with activity in some of these areas (insula, anterior cingulate). Furthermore, in pain, beta activity is increased in the insula and anterior cingulate [55], in accordance with this hypothesis.

On the other hand, in posterior cingulate based components no increased power is found. This might reflect the predominant autonomic-emotional-attentional aspects of distress (ACC based) and the limited influence of PCC based cognition [36], [56] Thus whereas the PCC is involved in tinnitus, as reflected by the ICA analysis, it seems it is not involved in tinnitus distress, but possibly forms part of a separable cognitive-memory related tinnitus network.

Contrary to expectation, no increased power is found in component 1 (Table 1), a dorsal ACC related network, extending to the insula, parahippocampus and DLPFC. As IC1 reflects an attentional network focusing on salient information it could be hypothesized that this network predominantly is involved in the intensity coding of the tinnitus. It has been shown that the intensity of perceived pain [57] and auditory [41] stimuli depends on fluctuations of activity in the dACC and anterior insula. The parahippocampal area, which acts as a sensory auditory gate [43], is involved in the percept of tinnitus [25], [58], as is the DLPFC [59], [60], [61]. As there is no significant difference in the perceived intensity in the different distress groups, this component will not differ between the different distress groups.

### Limitations of the study

One major limitation of this and any EEG based approach is that no subcortical activity can be analyzed, limiting network description to cortical sources. The data presented should therefore be viewed acknowledging this limitation. Another limitation of the present study relates to age differences between the tinnitus group and the control group. However, an analysis comparing age-matched tinnitus patients with low and high distress with respectively older patients with low and high distress showed no statistical differences. Furthermore, no prior research has shown that independent components change with age in resting state EEG, although power changes over age are expected. Secondly, as the control group was collected at a different lab it is possible that cultural background could also have an influence on our results. However, cross-cultural differences on independent components have not yet been shown across different continents [62]. Finally, the normative and clinical data have been acquired using different EEG machine, which may engender systematic distortions in the comparisons due to the different amplifiers response. However, we have analyzed relative measures, excluding confounding factors due to overall gain differences. On the other hand, frequency-specific distortions, if any, should appear in all components, which is not what we have observed. Hence, we think that differences demonstrated between both tinnitus group and the control group in our study might be reliable and valid, however further research is needed. This study demonstrates the need for a large normative database containing a large sample of all ages, and applicable to multiple EEG machines by calibration correction factors.

### Conclusion

Comparing patients with mild and very severe tinnitus distress to a normative BSS EEG database and comparing low with high distress permits to evaluate brain activity differences in functional networks associated with tinnitus distress. Based on this analysis it can be proposed that tinnitus distress results from alpha and beta abnormal activity in subgenual ACC extending to the pregenual and dorsal ACC and VM&VLPFC/OFC, insula, and parahippocampal area. This network overlaps partially with brain areas implicated in distress in patients suffering from pain, dyspnea, functional somatic syndromes and posttraumatic stress disorder, and might therefore represent an aspecific distress network.

## Materials and Methods

### Patients

Fifty-five tinnitus patients were selected from the multidisciplinary Tinnitus Research Initiative (TRI) Clinic of the University Hospital of Antwerp, Belgium (Table 3). The average age was 51 years (*SD* = 13). Individuals with pulsatile tinnitus, Ménière disease, otosclerosis, chronic headache, neurological disorders such as brain tumors, and individuals being treated for mental disorders were not included in the study in order to promote sample homogeneity. The patients selected for this study were not included in a previous study on tinnitus related distress conducted by the same research group [22].

**Table 3:** Tinnitus Characteristics.

		Grade		*statistic*
		Low distress	High distress	
Tinnitus laterality	*Left*	8	8	χ^2^ = .24, *n.s.*
	*Right*	7	6	
	*Bilateral*	15	11	
Tinnitus type	*Pure Tone*	7	8	χ^2^ = .52, *n.s.*
	*Narrow Band Noise*	23	17	
Tinnitus duration		*M* = 5.01	*M* = 5.10	*t* = −.10, *n.s.*
		*Sd* = 3.49	*Sd* = 3.32	
Tinnitus Intensity		*M* = 6.05	*M* = 6.27	*t* = −.66, *n.s.*
		*Sd* = 2.02	*Sd* = 1.90	
TQ		*M* = 32.90	*M* = 56.39	*t* = −.7.47, *n.s.*
		*Sd* = 12.57	*Sd* = 9.50	

*n.s.: not significant.*

All patients were investigated for the extent of hearing loss using audiograms. Tinnitus matching was performed looking for tinnitus pitch (frequency) and tinnitus intensity. Participants were requested to refrain from alcohol consumption 24 hours prior to recording, and from caffeinated beverages consumption on the day of recording.

Patients were also given the validated Dutch version of the Tinnitus Questionnaire [63], [64] originally published by Goebel and Hiller [65]. Goebel and Hiller described this TQ as a global index of distress and the Dutch version was further confirmed as a reliable measure for tinnitus-related distress [64]. Based on the total score on the TQ, participants were assigned to a low distress (0–46) points and high distress (47–84) category. Patient distribution in all groups for tinnitus laterality, tinnitus type, tinnitus duration and tinnitus intensity is represented in Table 2. No significant results were obtained between the two groups.

This study was approved by the local ethical committee (Antwerp University Hospital) and was in accordance with the declaration of Helsinki. We did not obtain an informed consent as this EEG recording was obtained for further diagnosis of the tinnitus patients and was a standard procedure for ongoing investigation.

### EEG data collection

EEG recordings (Mitsar-201, NovaTech http://www.novatecheeg.com/) were obtained in a fully lighted room with each participant sitting upright on a small but comfortable chair. The actual recording lasted approximately 5 min. The EEG was sampled with 19 electrodes (Fp1, Fp2, F7, F3, Fz, F4, F8, T7, C3, Cz, C4, T8, P7, P3, Pz, P4, P8, O1 O2) in the standard 10–20 International placement referenced to digitally linked ears, analogous to what is done in the normative group, and impedances were checked to remain below 5 kΩ. Data were collected eyes-closed (sampling rate  = 1024 Hz, band passed 0.15–200 Hz). Data were resampled to 128 Hz, band-pass filtered in the range 2–32 Hz and subsequently transposed into Eureka! software [66], plotted and carefully inspected for manual artifact-rejection. All episodic artifacts including eye blinks, eye movements, teeth clenching, body movement, or ECG artifact were removed from the stream of the EEG. We only removed episodic artifacts. Maximum 1 minute of artifact was removed. It is however difficult to say the number of artifacts that are removed for each patient as there is a relative large variability between patients.

### Normative database

Also the normative database of the Nova Tech EEG (NTE), Inc, Mesa, AZ (N = 84) was used (http://www.novatecheeg.com/). None of these subjects was known to suffer from tinnitus. Exclusion criteria for the NTE database were known psychiatric or neurological illness, psychiatric history of drug/alcohol abuse in a participant or any relative, current psychotropic/CNS active medications, history of head injury (with loss of consciousness) or seizures, headache, physical disability. To build the database about 3–5 min of EEG was continuously recorded while participant sat with the eyes closed on a comfortable chair in a quiet and dimly lit room. EEG data were acquired at the 19 standard leads prescribed by the 10–20 international system (FP1, FP2, F7, F3, FZ, F4, F8, T3, C3, CZ, C4, T4, T5, P3, PZ, P4, T6, O1, O2) using both earlobes as reference and enabling a 60 Hz notch filter to suppress power line contamination. The resistance of all electrodes was kept below 5 kΩ. Data of the NTE database were acquired using the 12-bit A/D NeuroSearch-24 acquisition system (Lexicor Medical Technology, Inc. Boulder, CO) and sampled at 128. The data were subsequently band-pass filtered in the region 2–32 Hz and artifact rejection was carried out using the same software and procedures as per the clinical data.

### sLORETA imaging

Standardized low-resolution brain electromagnetic tomography (sLORETA; Pascual-Marqui, 2002) was used to estimate the intracerebral electrical sources that generated the seven NICA components. As a standard procedure a common average reference transformation (see Pascual-Marqui [32]) is performed before applying the sLORETA algorithm. That is, the coordinates of the 19 electrode positions were applied to a digitized MRI version of the Talairach Atlas (McConnell Brain Imaging Centre, Montréal Neurological Institute, McGill University). These Talairach coordinates were then used to compute the sLORETA transformation matrix. More technical details can be found in [32].

sLORETA computes electric neuronal activity as current density (A/m2) without assuming a predefined number of active sources. The solution space used in this study and associated leadfield matrix are those implemented in the LORETA-Key software (freely available at URL http://www.uzh.ch/keyinst/loreta.htm).This software implements revisited realistic electrode coordinates (Jurcak et al. 2007) and the lead field produced by Fuchs et al. (2002) applying the boundary element method on the MNI-152 (Montreal neurological institute, Canada) template of Mazziotta et al. (2001). The sLORETA-key anatomical template divides and label the neocortical (including hippocampus and anterior cingulated cortex) MNI-152 volume in 6,239 voxels of dimension 5 mm^3^, based on probabilities returned by the Demon Atlas (Lancaster et al. 2000). The coregistration makes use of the correct translation from the MNI-152 space into the Talaiach and Tournoux (1988) space (Brett et al. 2002).

### Group Blind Source Separation

We employed the group blind source separation approach consisting in the approximate joint diagonalization of grand-average Fourier co-spectral matrices [31], [67]. Such method can separate uncorrelated sources with non-proportional power spectra [68] and is analogous to the averaging group ICA approach described for fMRI by Schmithorst and Holland [69]. Only co-spectra in the range of 2–32 Hz were diagonalized because in this band-pass region continuous EEG features the highest signal-to-noise ratio. Following previous work (Congedo et al., 2010) we extracted the seven most energetic components. The demixing matrix was used to extract the power of the seven normative components in both the normative sample and in the low and high distress group, as described in details in Congedo et al. [67], [70]. In addition, a group BSS analysis was conducted on the tinnitus group. We used the Aikake Information Criterium (AIC) to determine the number of components [71].

### Comparison between BSS components of normative database, low and high distressed tinnitus group

For each of the seven components (Table 1) relative power was computed with 1 Hz resolution with respect to the total energy across all components. Then the relative power for each frequency and each component was compared between the normative sample and the two Tinnitus groups (low and high distress). Multiple comparison Student-t tests were performed separately for each component. The significance threshold was based on a permutation test with 5000 permutations. The methodology used is non-parametric. It is based on estimating, via randomization, the empirical probability distribution for the max-statistic, under the null hypothesis [72]. This methodology corrects for multiple testing across frequencies and guarantees that the probability of falsely rejecting even only one hypothesis is less than the chosen alpha level.

### Correlation analysis

A correlation analysis was conducted between the relative power of the seven components and the TQ-scores. TQ-scores are used and not the TQ grade to have a continuous variable that can be correlated to the specific independent component. This analysis has as advantage that the tinnitus group is not divided in to two separate groups (low vs high distress). Analysis was performed in all 4 Hz spaced discrete Fourier frequencies in the range 2–32 Hz (2–4 Hz, 4–8 Hz, 8–12 Hz, 12–16 Hz, 16–20 Hz, 20–24 Hz, 24–28 Hz, 28–32 Hz). Corrections were performed for multiple comparisons across eight frequencies bands using a Bonferroni method and testing separately for each component.

### Functional Connectivity

Functional connectivity between time series corresponding to different spatial locations is calculated using lagged coherence [73]. Based on the method introduced by Pascual-Marqui, this measure of dependence can be applied to any number of brain areas jointly, that is, they reflect a global functional connectivity between all series included in the analysis. Time-series were extracted for different ROIs using sLORETA. The measures are non-negative and take the value zero only when there is independence. They were defined in the frequency domain in the range 2–32 Hz (2–4 Hz, 4–8 Hz, 8–12 Hz, 12–16 Hz, 16–20 Hz, 20–24 Hz, 24–28 Hz, 28–32 Hz). Regions of interest were defined based upon the areas involved in IC5 and IC6 (Table 2).

## Supporting Information

Figure S1 **Comparison for the independent components C5 generated from the normative database (middle) and compared with an aged-matched and older tinnitus group.** Left and right panels: Relative Power (arbitrary units) of component along frequencies in the range 2–32 Hz for low distress (left) and high distress (right) in tinnitus patients. Black solid line represents the mean, dotted black lines 95% confidence intervals. Pink dots represent statistically significant (p<0.05) increased power, plotted for each frequency (on X-axis) and the relative power on the Y-Axis.
(TIF)

Figure S2 **Comparison for the independent components C6 generated from the normative database (middle) and compared with an aged-matched and older tinnitus group.** Left and right panels: Relative Power (arbitrary units) of component along frequencies in the range 2–32 Hz for low distress (left) and high distress (right) in tinnitus patients. Black solid line represents the mean, dotted black lines 95% confidence intervals. Pink dots represent statistically significant (p<0.05) increased power, plotted for each frequency (on X-axis) and the relative power on the Y-Axis.
(TIF)

Figure S3 **Scatterplots for respectively the alpha frequency band (8–12 Hz) and the beta frequency band (12–20 Hz) between TQ and the relative power for IC5.**
(TIF)

Figure S4 **Scatterplot for respectively the alpha frequency band (8–12 Hz) and the beta frequency band (12–26 Hz) between TQ and the relative power for IC6.**
(TIF)

Figure S5 **Scatterplots for respectively the alpha frequency band (8–12 Hz) and the beta frequency band (12–24 Hz) between TQ and the relative power for Tinnitus IC4.**
(TIF)

Table S1 **Young and Old Tinnitus patients.**
(DOCX)
