# Supplementary figures and images for: The Distressed Brain: A Group Blind Source Separation Analysis on Tinnitus
Source: PLoS One. 2011 Oct 6;6(10):e24273. doi: 10.1371/journal.pone.0024273 (PMC3188549; doi:10.1371/journal.pone.0024273)

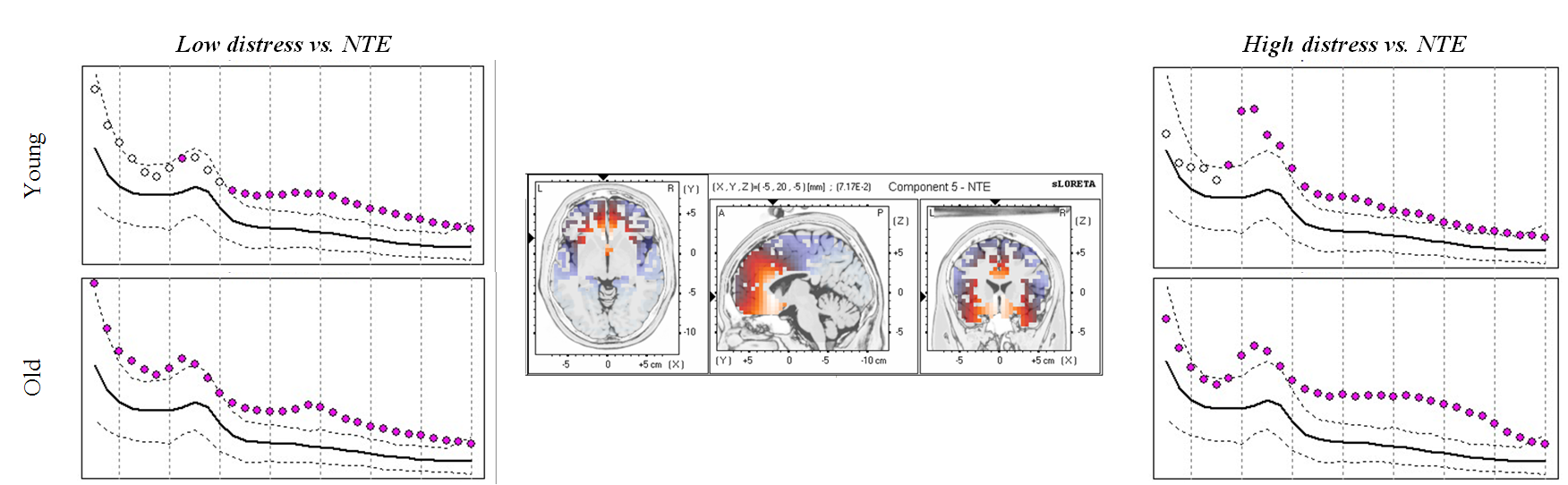

Supplement: Figure S1 — Comparison for the independent components C5 generated from the normative database (middle) and compared with an aged-matched and older tinnitus group. Left and right panels: Relative Power (arbitrary units) of component along frequencies in the range 2–32 Hz for low distress (left) and high distress (right) in tinnitus patients. Black solid line represents the mean, dotted black lines 95% confidence intervals. Pink dots represent statistically significant (p<0.05) increased power, plotted for each frequency (on X-axis) and the relative power on the Y-Axis. (TIF) [file pone.0024273.s001.tif]

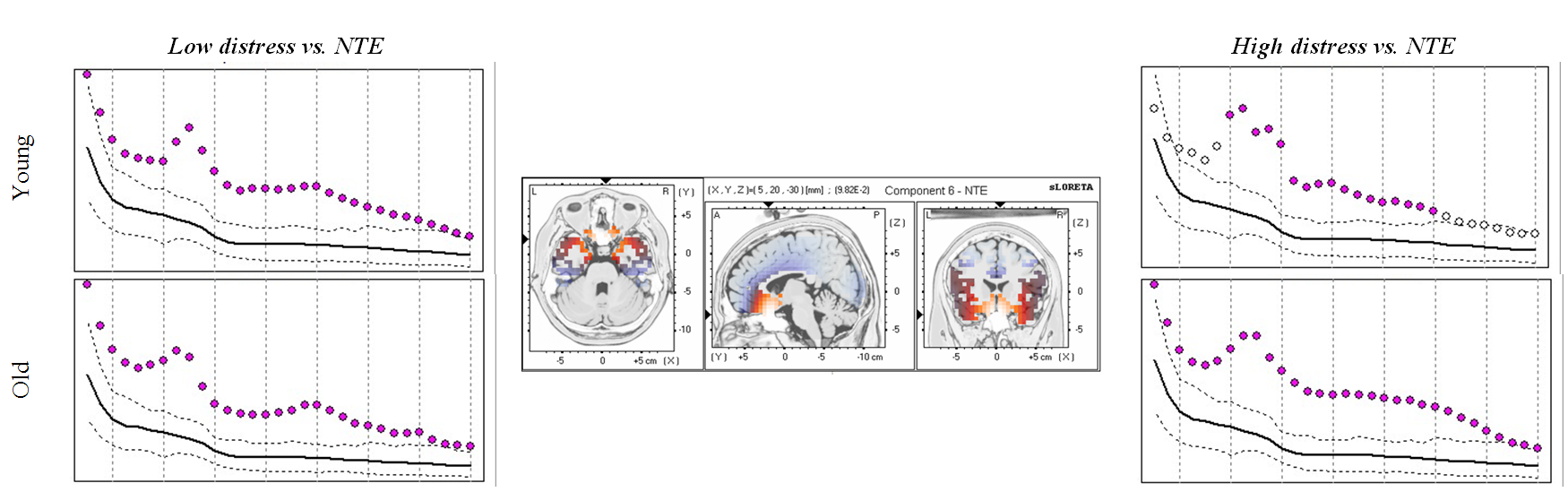

Supplement: Figure S2 — Comparison for the independent components C6 generated from the normative database (middle) and compared with an aged-matched and older tinnitus group. Left and right panels: Relative Power (arbitrary units) of component along frequencies in the range 2–32 Hz for low distress (left) and high distress (right) in tinnitus patients. Black solid line represents the mean, dotted black lines 95% confidence intervals. Pink dots represent statistically significant (p<0.05) increased power, plotted for each frequency (on X-axis) and the relative power on the Y-Axis. (TIF) [file pone.0024273.s002.tif]

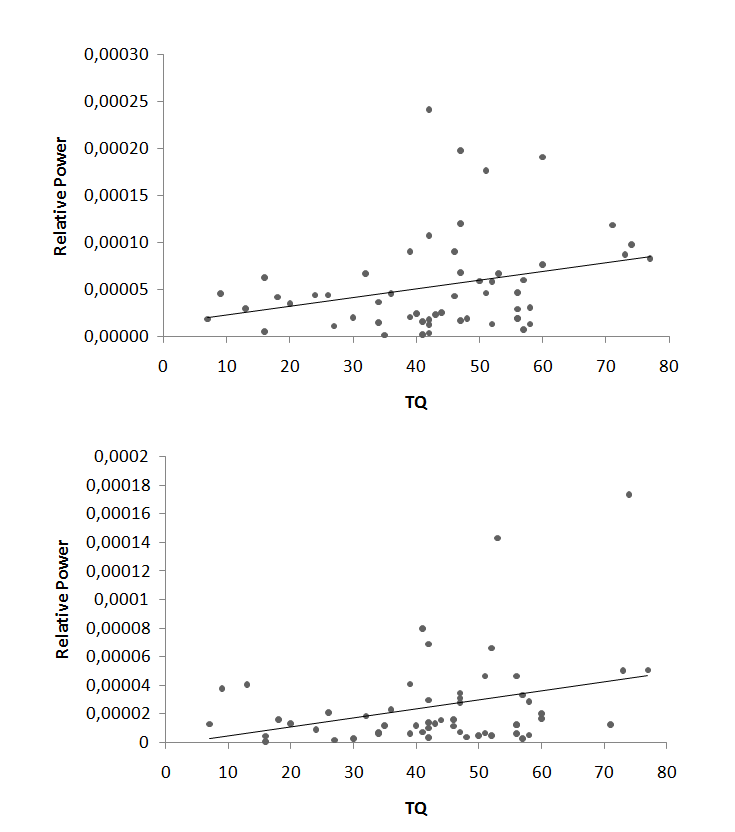

Supplement: Figure S3 — Scatterplots for respectively the alpha frequency band (8–12 Hz) and the beta frequency band (12–20 Hz) between TQ and the relative power for IC5. (TIF) [file pone.0024273.s003.tif]

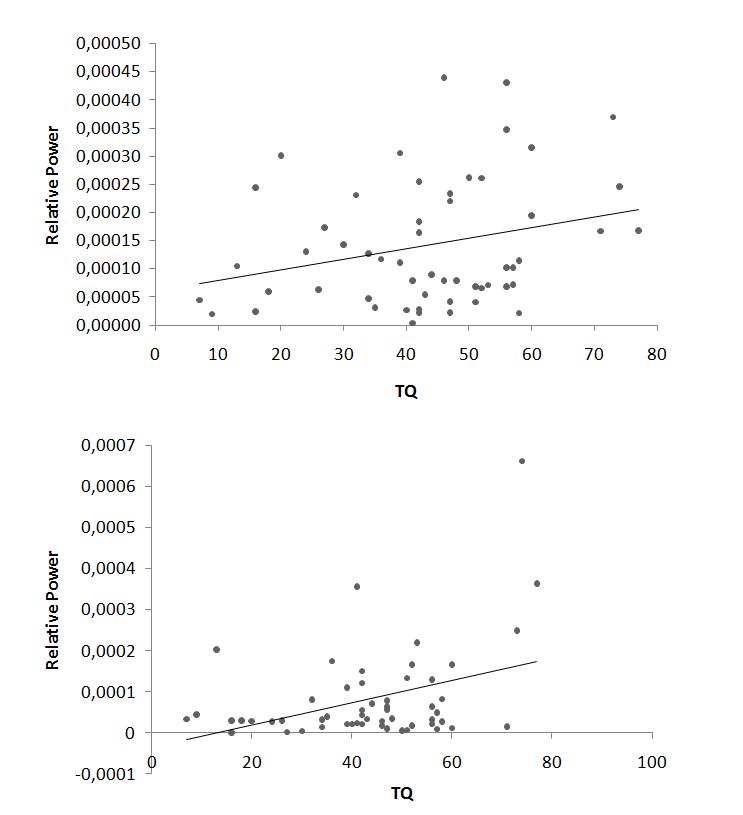

Supplement: Figure S4 — Scatterplot for respectively the alpha frequency band (8–12 Hz) and the beta frequency band (12–26 Hz) between TQ and the relative power for IC6. (TIF) [file pone.0024273.s004.tif]

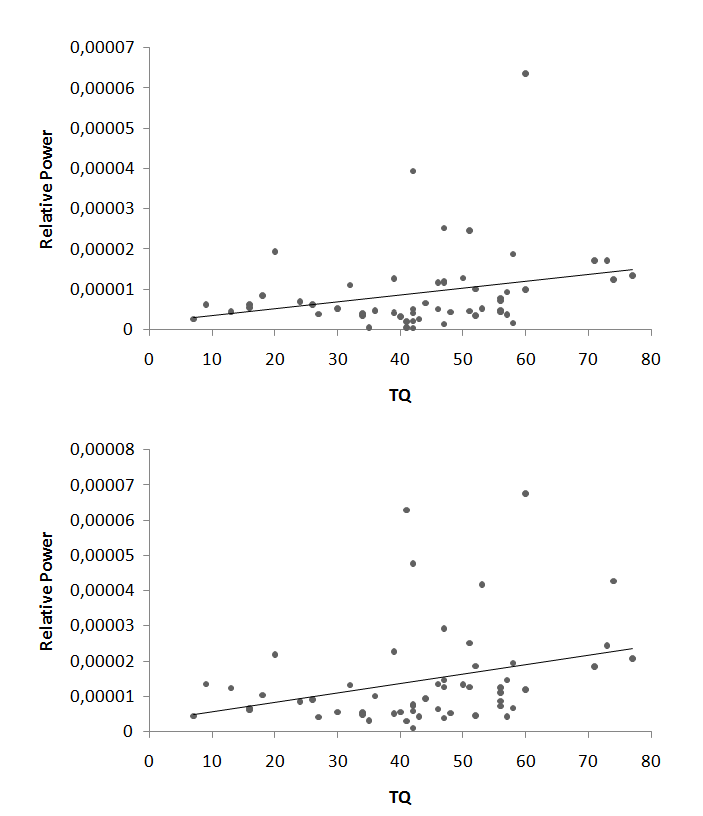

Supplement: Figure S5 — Scatterplots for respectively the alpha frequency band (8–12 Hz) and the beta frequency band (12–24 Hz) between TQ and the relative power for Tinnitus IC4. (TIF) [file pone.0024273.s005.tif]
