# Supplementary material for: The Distressed Brain: A Group Blind Source Separation Analysis on Tinnitus
Source: PLoS One. 2011 Oct 6;6(10):e24273. doi: 10.1371/journal.pone.0024273 (PMC3188549; doi:10.1371/journal.pone.0024273)
Supplement: Table S1 — Young and Old Tinnitus patients. (DOCX) [file pone.0024273.s006.docx]

**Table S1. Young and Old Tinnitus patients**

|  | **Grade** | | | |
| --- | --- | --- | --- | --- |
|  | **Low distress** | | **High distress** | |
|  | Young | Old | Young | Old |
| Gender | 7 | 21 | 6 | 19 |
| Age | *M* = 30.40 | *M* = 58.30 | *M =*31.06 | *M* = 57.79 |
